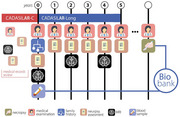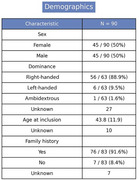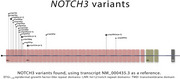# CADASIL Argentine Registry: Study Design and Preliminary Data

**DOI:** 10.1002/alz70857_101610

**Published:** 2025-12-25

**Authors:** Carolina Agata Ardohain Cristalli, Julieta Rosales, Fabio Gonzalez, Valentin Selvaggi, Julián Martín Alonso, Juan Ignacio López, Martín Aguilar, Marcelo Kauffman, Danit G Saks, Ricardo Allegri, Gustavo Sevlever, Hernan Chaves, Diana Olga Cristalli, Ismael Luis Calandri

**Affiliations:** ^1^ Fleni, Buenos Aires, Buenos Aires, Argentina; ^2^ La Sagrada Familia, CABA, Buenos Aires, Argentina; ^3^ Hospital Británico, CABA, Buenos Aires, Argentina; ^4^ Hsopital Ramos Mejía, CABA, Buenos Aires, Argentina; ^5^ Hospital Posadas, CABA, Buenos Aires, Argentina; ^6^ Sanatorio Los Arcos, CABA, Buenos Aires, Argentina; ^7^ Fleni, CABA, Buenos Aires, Argentina; ^8^ Hospital Ramos Mejía, CABA, Buenos Aires, Argentina; ^9^ Centre for Healthy Brain Ageing (CHeBA), University of New South Wales, Sydney, NSW, Australia; ^10^ Fleni, Buenos Aires, Argentina; ^11^ Centro Jesi, La Plata, Buenos Aires, Argentina

## Abstract

**Background:**

Cerebral autosomal dominant arteriopathy with subcortical infarcts and leukoencephalopathy (CADASIL), the most common hereditary small vessel disease, leads to early‐onset stroke and vascular cognitive impairment (VCI). Despite its importance, data from Latin America remain scarce. The CADASIL Argentine registry (CADASILAr) was created to harmonize clinical data, promote international collaboration, and provide a reproducible, longitudinal framework to study disease progression and expand to neighboring countries. This study aims to present the cohort design and preliminary results from the cross‐sectional phase.

**Method:**

CADASILAr was developed to document demographic, clinical, imaging, and genetic features of CADASIL patients and to explore factors associated with disease progression and cognitive decline in an Argentinian multisite cohort. The study includes two phases: (1) a cross‐sectional phase (CADASILAR‐C) and (2) a longitudinal phase (CADASILAR‐Long), following adults aged ≥18 years with genetically confirmed or suspected CADASIL. Variables collected include demographics, symptom onset, clinical features, neuroimaging, genetic data, and vascular risk factors. The study also examines socio‐economic disparities, integrates biobanks, and harmonizes data collection with international CADASIL and dementia registries. Longitudinal follow‐ups are planned annually over 5 years (Figure 1), with cognitive batteries aligned with international cohorts and a brain donation program to establish a CADASIL brain bank in Argentina.

**Result:**

Preliminary data from 90 patients (50% female) show a mean age of 43.8 ± 11.9 years, with family history in 91.6% (Figure 2). The most common clinical presentations were cerebrovascular events (72.9%), cognitive impairment (56.7%), and migraine (69%). The most frequent comorbidities included hypertension (64%) and dyslipidemia (55%). Among 86 confirmed cases, 63 were diagnosed through genetic testing and 20 through skin biopsy. Genetic analysis identified cysteine‐altering NOTCH3 mutations in all confirmed cases, predominantly affecting epidermal growth factor‐like repeats (Figure 3). Of the 33 patients assessed with the MMSE, the median score was 28 (IQR: 22–29).

**Conclusion:**

CADASILAr is the first systematic effort to study this disease in Latin America and the twelfth global CADASIL registry. By integrating baseline and longitudinal data, it offers a robust platform to investigate genetic, neuroimaging, and cognitive outcomes while fostering international collaborations to advance research and understanding of CADASIL.